# Goniometric Assessment in French Bulldogs

**DOI:** 10.3389/fvets.2019.00424

**Published:** 2019-12-13

**Authors:** Maira Rezende Formenton, Lidiane Gonçalves de Lima, Flávia Gardilin Vassalo, Jean Guilherme Fernandes Joaquim, Laryssa Petrocini Rosseto, Denise Tabacchi Fantoni

**Affiliations:** ^1^Department of Surgery, School of Veterinary Medicine and Animal Science, University of São Paulo, São Paulo, Brazil; ^2^Veterinary Practitioner, Ribeirao Preto, Brazil; ^3^Physical Therapy Department, Instituto Bioethicus, Botucatu, Brazil; ^4^FisioPet, Ribeirao Preto, Brazil

**Keywords:** canine, goniometry, small animals, physical therapy, range of motion, dogs

## Abstract

Goniometry is a low-cost, user-friendly and widely available technique used by different veterinary medicine professionals to estimate joint range of motion (ROM). Studies providing breed-specific reference ranges for goniometric measurements are scarce and there is a lack of information regarding joint angles in French Bulldogs. This prospective study set out to determine normal ROM for the carpus, elbow, shoulder, tarsus, stifle and hip joints in healthy, adult French Bulldogs using goniometry. We hypothesized ROM would be similar in this and other dog breeds. Twenty dogs met the inclusion criteria. Sample size was calculated using power analysis based on previous studies. Goniometric measurements were made by a single examiner. Limbs were measured in random order and three measurements made per joint. Dogs were not sedated. Joint angles measured in French Bulldogs in this study were similar to those reported in Labrador Retrievers (shoulder, carpal, and tarsal flexion), Rottweilers (shoulder, carpus, and hip flexion), and Dachshunds (hip, stifle, and tarsal flexion). Similar flexion angles and ROM were detected in right and left limb joints. Findings of this study suggest similar ROM in French Bulldogs and other dog breeds. Lack of radiographic assessment and the fact that goniometric measurements were made by a single examiner were the major limitations of this study.

## Introduction

Goniometric measurement of joint angles is widely used by orthopedic surgeons and physical therapists to estimate joint range of motion (ROM). Goniometry is a static, low-cost, user-friendly method, and an extremely efficient and reliable ROM assessment tool (1–3). It is also thought to be a useful technique for routine monitoring of patient progression and response to physical rehabilitation, given the close relationship between decreased joint angles and joint stiffness in osteoarthritic patients (1, 4). There is a great need to determine breed-specific ROM in dogs (5, 6), as related literature is limited to a few studies in Labrador retrievers (2), German shepherds (7), and Rottweilers (8).

French Bulldogs have recently enjoyed increasing popularity among brachycephalic breeds (9). Kyphosis and several vertebral malformations have been reported in dogs of this breed, with significant clinical and body conformation implications (10). Still, popular as French Bulldogs may be, joint angles have not been quantified in this breed.

Joint angles can be measured with animals in the lateral recumbent or standing position, via manipulation of thoracic and pelvic limb joints (i.e., passive joint flexion, extension, abduction and adduction, and measurement of joint angles achieved during these movements) (2, 7). Goniometric assessment includes measurements of shoulder flexion and extension, as well as elbow, carpus, hip, stifle and tarsus flexion and extension (11, 12). This study set out to determine normal ROM of the shoulder, elbow, carpus, hip, stifle and tarsus joints in healthy, non-sedated French Bulldogs using goniometry.

## Methods

This study was approved by the Ethics Committee for Animal Use of the School of Veterinary Medicine and Animal Science, University of São Paulo, protocol No. 1571120219. Patients were recruited from five different cities located in the state of São Paulo, Brazil. Patient selection was based on clinical history and evaluation, including inspection, joint palpation and ancillary tests. Inclusion criteria were as follows: male or female adult dogs aged 16–48 months, with body condition score ranging from 4 to 6 on a 1-to-9 scale (13). Owners were interrogated as to history of orthopedic conditions or trauma. Animals were inspected in the standard anatomical position for signs of musculoskeletal changes, pain or asymmetries. Subjective muscle asymmetry findings were assessed using perimetric measurements taken with a measuring tape. Clinical and gait assessment at the walk and trot were carried out to search for lameness and joint abnormalities; these were followed by joint palpation in full ROM for signs of crepitus, effusion or instability. Patients were also submitted to specific orthopedic tests, such as the tibial compression (cranial tibial thrust), the patellar luxation and the Ortolani test (14).

Seven exclusion criteria (EC) were defined to ensure the selection of dogs with no apparent musculoskeletal dysfunction that might interfere with goniometric assessment, as follows:

• EC1: Signs of lameness, joint noises and/or limb rotation at the walk and/or trot.

• EC2: Presence of atrophy, asymmetries, or musculoskeletal changes confirmed by perimetric measurement.

• EC3: Joint crepitation, edema, or instability on palpation.

• EC4: Pain manifestations during clinical evaluation.

• EC5: Pregnancy.

• EC6: Positive tibial compression (cranial tibial thrust) test.

• EC7: Positive Ortolani test.

Goniometric assessment was performed with dogs lying on the examination table. Dogs were manually restrained with their owner's assistance; no sedation was required. A plastic 35 cm, 0° to 180° system universal goniometer with two-degree increments (Carci®, São Paulo-SP, Brazil) was used. Dogs were placed in left or right lateral recumbency and the goniometer positioned according to instructions given previous authors (2, 15). Measurements were made by a single examiner (animal rehabilitation specialist with 3 years of professional experience). Range of motion was measured in flexion and extension; three measurements were made per joint for increased accuracy. Limb order was randomly selected using Random Number Generator App.

**Figure 1:**
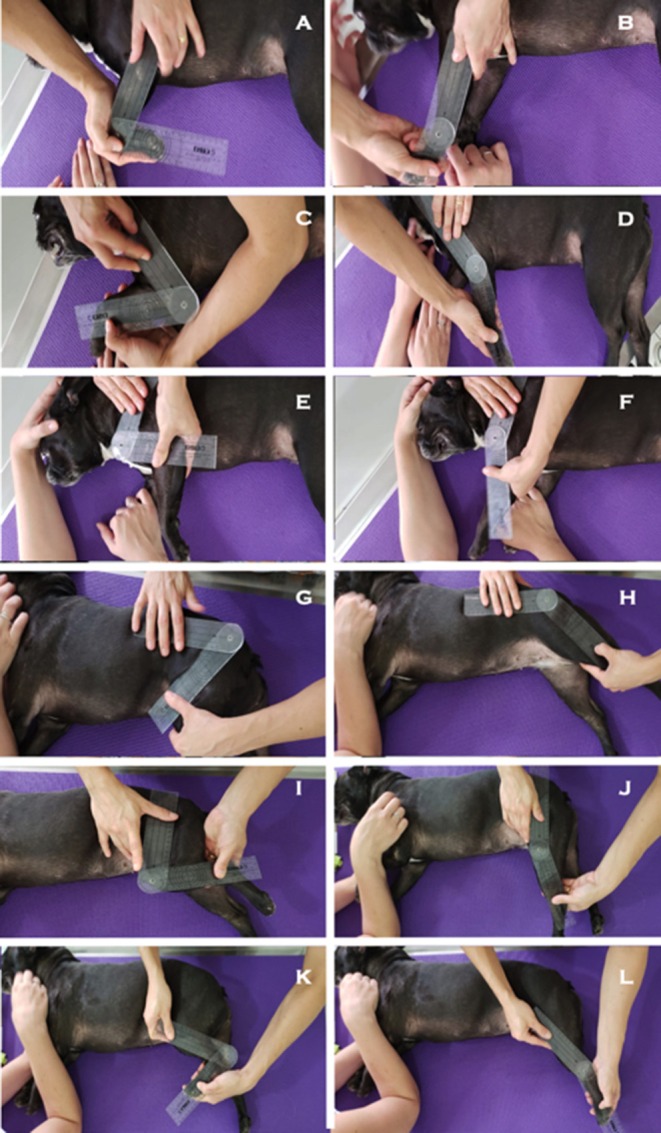
Correct goniometer positioning **(A)** carpal flexion; **(B)** carpal extension; **(C)** elbow flexion; **(D)** elbow extension; **(E)** shoulder flexion; **(F)** shoulder extension; **(G)** hip flexion; **(H)** hip extension; **(I)** stifle flexion; **(J)** stifle extension; **(K)** tarsal flexion; and **(L)** tarsal extension.

Shoulder, elbow, carpus, hip, stifle, and tarsus ROM were measured with joints in maximum flexion and extension. Data were analyzed using Google Sheets software. Arithmetic means, standard deviations (SD) and coefficients of variation (CV) were calculated for statistical analysis. Minimum sample size (6 animals) was determined by power analysis (*p* < 0.05; 95%) based on carpal and hip extension measurements derived from similar trials (2) and a pilot study. Correct placement of the goniometer is shown in Figure 1. Anatomical landmarks used as reference points for goniometer positioning are described in Table 1.

**Table 1:** Anatomical landmarks used as reference points for goniometer positioning prior to joint flexion and extension angle measurements, according to instructions given elsewhere (2, 15).

**Joint**	**Position of goniometer center and arms**
Carpus	The center of the goniometer was placed over the axis of joint rotation. One arm of the goniometer was placed along the long axis of metacarpal bones III and IV and the other one along the longitudinal axis of the antebrachium
Elbow	The center of the goniometer was placed over the axis of joint rotation. One arm of the goniometer was placed along the longitudinal axis of the antebrachium and the other one along the longitudinal axis of the humerus
Shoulder	The center of the goniometer was placed over the axis joint of rotation. One arm of the goniometer was placed along the longitudinal axis of the humerus and the other one along the spine of the scapula
Tarsus	The center of the goniometer was placed over the axis joint of rotation. One arm of the goniometer was placed along the longitudinal axis of metatarsal bones III and IV and the other one along the tibial shaft
Stifle	The center of the goniometer was placed over the axis of joint rotation. One arm of the goniometer was placed along the tibial shaft and the other one along the longitudinal axis of the femur
Hip	The center of the goniometer was placed over the axis of joint rotation. One arm of the goniometer was placed along the longitudinal axis of the femur and the other one along a line joining the tuber sacrale and tuber ischiadicum

## Results

Thirty-seven dogs were evaluated; of these, 17 (46%) met one or more EC; the final sample comprised 20 dogs (54%; 5 males and 15 females). Joint angle and ROM data of dogs in this sample are shown in Table 2.

**Table 2:** Mean maximum flexion and extension angles and range of motion of right and left thoracic and pelvic limb joints of French Bulldogs, and respective standard deviations (SD) and coefficients of variation (CV).

**Joint**	**Extension**		**Flexion**		**Range of motion**	
	**Right limb**	**Left limb**	**Right limb**	**Left limb**	**Right limb**	**Left limb**
	**Mean ± SD**	**Mean ± SD**	**Mean ± SD**	**Mean ± SD**	**Mean ± SD**	**Mean ± SD**
	**CV**	**CV**	**CV**	**CV**	**CV**	**CV**
Shoulder	160 ± 19	160 ± 20	51 ± 8	52 ± 9	109 ± 24	108 ± 24
	12.09%	12.34%	16.29%	17.50%	21.84%	21.77%
Elbow	174 ± 11	175 ± 9	51 ± 13	49 ± 12	123 ± 18	126 ± 16
	6.24%	5.00%	24.67%	23.98%	14.63%	12.78%
Carpus	204 ± 8	204 ± 8	32 ± 7	32 ± 7	172 ± 10	172 ± 10
	4.03%	4.01%	22.69%	21.53%	5.54%	5.75%
Hip	181 ± 7	179 ± 7	58 ± 10	59 ± 10	123 ± 14	121 ± 14
	4.14%	4.07%	17.47%	16.84%	11.05%	11.23%
Stifle	172 ± 8	174 ± 8	58 ± 8	59 ± 10	114 ± 12	115 ± 12
	4.72%	4.33%	14.22%	17.02%	10.28%	10.70%
Tarsus	188 ± 7	188 ± 6	40 ± 6	39 ± 7	149 ± 8	149 ± 9
	3.74%	3.05%	16.33%	16.60%	5.36%	5.76%

Coefficients of variation express standard deviation as a percentage of the average and may be low, medium or high (>10%, 10–20%, and 20–30%, respectively). Coefficients of variation above 30% are thought to be too high to ensure data quality. In this study, CV values fell within the low, medium or high ranges and did not exceed 30%. Therefore, this data set was deemed homogeneous. Also, comparative analysis of right and left side measurements revealed similar angles, suggesting symmetrical muscle thickness, and ROM overall.

## Discussion

This study described normal ROM of the shoulder, elbow, carpus, hip, stifle and tarsus joints in healthy, non-sedated French Bulldogs based on goniometric measurements. Shoulder extension angles in this study were similar to those reported in Labrador Retrievers (2) and cats (16), whereas shoulder, carpal, and tarsal flexion angles reflected those reported in Labrador Retrievers (2). Shoulder, carpus, and hip flexion angles were also comparable to ranges reported in Rottweilers (8). Range of motion was similar in left and right limbs. Full shoulder extension movements are thought to be uncommon in dogs (2). This may explain conflicting findings in this study and the reluctance of some animals to extend their shoulders. Dogs in this sample did not show signs of pain; however, arthritis or other joint/bone abnormalities cannot be ruled out, as radiographic assessment was not performed.

According to Freund et al. (3), radiographic and goniometric measurements of the canine stifle differ. However, radiographic assessment of joint angles is uncommon and goniometric measurement in lateral recumbency is the method of choice in routine practice of physical therapy (3, 4).

Studies comparing goniometric and radiographic measurements in non-sedated and sedated dogs failed to reveal significant impacts of sedation on radiographic measurements of joint angle (2). French Bulldogs in this study did not require sedation given their docile temperament.

Findings of this study are in keeping with data reported in a goniometric study evaluating hip, stifle and tarsal flexion angles in Dachshunds (17). In that study (17) pelvic limb muscle mass was thought to make landmarks for goniometer placement particularly difficult to palpate compared to long-legged dogs. Precise location of anatomical landmarks mitigates problems associated with placement of a flat goniometer on chunky, curvy limbs.

In this study, standard deviations, and coefficients of variation were calculated for improved accuracy of angle estimates (3, 7, 18). Coefficients of variation express standard deviation as a percentage of the average and may be low, medium or high (>10%, 10–20%, and 20–30%, respectively). Coefficients of variation above 30% are thought to be too high to ensure data quality. In this study, CV values fell within the low, medium or high ranges and did not exceed 30%. Therefore, this data set was deemed homogeneous. Also, comparative analysis of right and left side measurements revealed similar angles, suggesting symmetrical muscle thickness and ROM overall.

Comparison of findings reported by different investigators is thought to add reliability to research data (2, 17). Similar goniometric studies in dogs of the French Bulldog breed are lacking, therefore no comparisons could be made. Goniometric data collection by multiple examiners is also recommended. However, goniometric measurements made by different experienced examiners are not thought to be significantly different (2) and manipulation by a single examiner may minimize stress levels in canine patients. Also, the single examiner in this study does have the advantage of consistency in technique.

Major limitations of this study include: lack of radiographic confirmation of joint/limb soundness, lack of comparative radiographic measurements of ROM and technical difficulties associated with goniometric measurements in dogs with well-developed pelvic and thoracic limb muscles such as French Bulldogs.

## Conclusion

This study revealed symmetrical ROM in left and right pelvic and thoracic limb joints in French Bulldogs. Similar ROM in French Bulldogs and other dog breeds support the reliability of data collected in this study. However, assessment by more than one examiner and the inclusion of radiographic measurements might have further increased data accuracy. Findings of this study will benefit future studies and practitioners who are rehabilitating French Bulldogs.

## Data Availability Statement

All datasets generated for this study are included in the article/supplementary material.

## Ethics Statement

The animal study was reviewed and approved by University of São Paulo. Written informed consent was obtained from the owners for the participation of their animals in this study.

## Author Contributions

MF contributed with study design, writing, and review. LL contributed to the goniometric assessment of the dogs. FV and LR contributed with study design, writing, and pictures. JJ and DF contributed to review.

### Conflict of Interest

FV and JJ currently work for Bioethicus Institute; LR works for FisioPet. The remaining authors declare that the research was conducted in the absence of any commercial or financial relationships that could be construed as a potential conflict of interest.
